# The Baculovirus Uses a Captured Host Phosphatase to Induce Enhanced Locomotory Activity in Host Caterpillars

**DOI:** 10.1371/journal.ppat.1002644

**Published:** 2012-04-05

**Authors:** Susumu Katsuma, Yasue Koyano, WonKyung Kang, Ryuhei Kokusho, Shizuo George Kamita, Toru Shimada

**Affiliations:** 1 Department of Agricultural and Environmental Biology, Graduate School of Agricultural and Life Sciences, The University of Tokyo, Yayoi, Bunkyo-ku, Tokyo, Japan; 2 Molecular Entomology Laboratory, RIKEN, Hirosawa, Wako, Japan; 3 Department of Entomology, University of California, Davis, California, United States of America; Stanford University, United States of America

## Abstract

The baculovirus is a classic example of a parasite that alters the behavior or physiology of its host so that progeny transmission is maximized. Baculoviruses do this by inducing enhanced locomotory activity (ELA) that causes the host caterpillars to climb to the upper foliage of plants. We previously reported that this behavior is not induced in silkworms that are infected with a mutant baculovirus lacking its protein tyrosine phosphatase (*ptp*) gene, a gene likely captured from an ancestral host. Here we show that the product of the *ptp* gene, PTP, associates with baculovirus ORF1629 as a virion structural protein, but surprisingly phosphatase activity associated with PTP was not required for the induction of ELA. Interestingly, the *ptp* knockout baculovirus showed significantly reduced infectivity of larval brain tissues. Collectively, we show that the modern baculovirus uses the host-derived phosphatase to establish adequate infection for ELA as a virion-associated structural protein rather than as an enzyme.

## Author Summary

Pathogens are known to usurp or alter the behavior of their hosts for their own benefit. Such behavior modification by animal and even plant viruses is widely observed in insect hosts. One of the earliest documented examples of such behavior modification is *Wipfelkrankheit*, a baculovirus-induced disease that causes caterpillars to migrate to the upper foliage of food plants where they die. Two baculovirus genes, *ptp* and *egt*, are involved in the induction of enhanced locomotory activity (ELA) such as climbing behavior in baculovirus-infected caterpillars. Here we dissect the functional role that baculovirus protein tyrosine phosphatase (PTP), the protein encoded by *ptp*, plays in the induction of ELA. We surprisingly found that PTP functions as a virus-associated structural protein and not as an enzyme in regard to the induction of ELA. We show that PTP plays a crucial role in virus infection of brain tissues, and hypothesize that this infection results in pathogen control of insect behavior. Since *ptp* was likely captured from an ancestral host by horizontal gene transfer, our findings tell an amazing story of how the modern baculovirus uses a captured host gene in a completely different way from how it was likely used in the ancestral host.

## Introduction

Viruses and other parasites are known to usurp or alter the behavior of their hosts for their own benefit. This type of behavior modification by animal and even plant viruses is widely observed in arthropod hosts [1], [2]. One of the earliest documented examples of such behavior modification is *Wipfelkrankheit* or tree-top disease of caterpillars [3]. A hallmark of this disease is enhanced locomotory activity (ELA) that causes the diseased caterpillars to migrate to the upper foliage of the host plant where they die. We now know that the causative agent of *Wipfelkrankheit* is a large, double-stranded DNA virus in the family Baculoviridae. Baculoviruses form a large group of arthropod-specific pathogens that commonly attack lepidopteran insects [4]. The baculovirus genome is large, 80 to over 160 kbp, and generally encodes more than 100 potential genes of which more than 10% appear to be derived from an ancestral host [5]. Baculoviruses produce two types of progeny during their infection cycle: the budded virus (BV) and occlusion-derived virus (ODV). BVs are involved in spread of the virus within an infected host. ODVs on the other hand are occluded within an occlusion body (OB) that protects and transmits the ODV from insect-to-insect via oral infection [6], [7].

At a late stage of infection, baculovirus-infected lepidopteran larvae often display ELA [3], [8], [9] and climb to the top of the host plant where they die and liquefy after death. It is believed that this behavior results in the dispersal of progeny OBs over a larger surface area thus improving the chance of virus transmission to other hosts. We have previously identified a protein tyrosine phosphatase (*ptp*) gene of the baculovirus, *Bombyx mori* nucleopolyhedrovirus (BmNPV) that induces wandering-like ELA in the silkworm *B. mori*. This gene was identified by behavioral screening of silkworms against a library of gene knockout mutants of BmNPV. Interestingly, the BmNPV *ptp* gene appears to have been acquired by an ancestral BmNPV from an ancestral silkworm [8]. Unlike silkworms that are infected with wild-type BmNPV, silkworms that are infected with a *ptp*-deleted BmNPV do not show ELA. The protein encoded by *ptp*, PTP, shows dephosphorylation activity with protein and RNA as substrate [10]–[13], however, the role that PTP plays in the induction of ELA is still unknown. Recently, a knockout mutant of the baculovirus *Lymantria dispar* nucleopolyhedrovirus (LdMNPV) has also been shown to exhibit reduced ELA in comparison to wild-type LdMNPV in the European gypsy moth [9]. Specifically, gypsy moths infected with an *ecdysteroid UDP-glucosyltransferase* (*egt*) gene deletion mutant of LdMNPV show reduced climbing behavior. Here we surprisingly show that baculovirus PTP induces ELA as a structural protein, and not as an enzyme. Furthermore, we show that baculovirus infection of brain tissues appears to be important for the induction of ELA.

## Results

### BmNPV-induced ELA in larval *B. mori* requires PTP protein but not PTP-associated phosphatase activity

**Figure 1:**
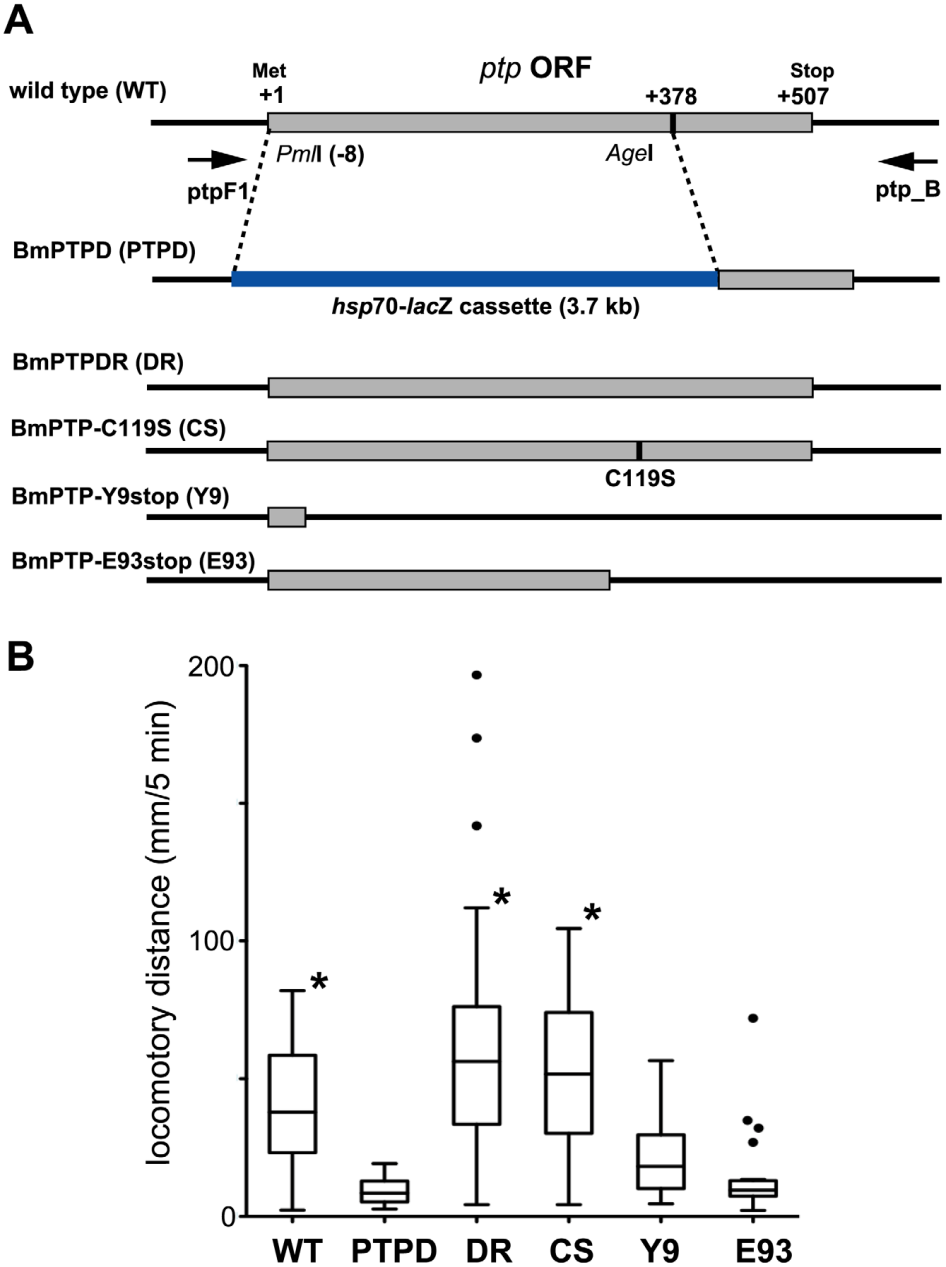
Effect of mutation of the BmNPV *ptp* gene on virus-induced ELA in 5^th^ instar *B. mori*. (A) Schematic representation of the *ptp* gene locus of wild-type (WT) and mutant BmNPVs. The locations of PCR primers (ptpF1 and ptp_B) used in the genotyping experiments are represented by the arrows. In BmPTPD (PTPD), nucleotides −8 to 377 of the *ptp* gene are replaced by a *hsp*70-*lac*Z gene cassette (3.7 kbp). BmPTPDR (DR) is a repair mutant in which the *hsp*70-*lac*Z gene cassette of BmPTPD was replaced with the original BmNPV sequence. BmPTP-C119S (CS) contains a point mutation within the *ptp* gene which results in an amino acid residue substitution (C119S) within the predicted P-loop motif that is required for phosphatase activity. BmPTP-Y9stop (Y9) and BmPTP-E93stop (E93) contain point mutations within the *ptp* gene which generate stop codons at Tyr-9 and Glu-93, respectively, but the RNA structures of the respective transcripts are likely to be unchanged. (B) Induction of ELA in 5^th^ instar *B. mori* injected with wild-type or mutant BmNPVs. Distances traveled at 90 h p.i. are shown by *box-and-whisker diagrams*. The boxes represent the median and 25–75 percentile ranges of the distances traveled. The w*hiskers* indicate the most extreme data points, which were no more than 1.5 times the interquartile range from the *boxes*. The dots indicate outliers predicted by Prism software. BmPTPD did not induce any ELA during the assay period. *p<0.05, Kruskal-Wallis analysis with Dunn's post test in comparison to the value obtained for BmPTPD. The abbreviations of the viruses are the same as in A.

We previously reported that a *ptp* gene deletion mutant of BmNPV (BmPTPD) does not induce ELA in larval *B. mori* at a late stage of infection [8]. This suggested that baculovirus-induced ELA involves the dephosphorylation of an unknown protein or RNA target by baculovirus PTP. To test this hypothesis, we generated BmPTP-C119S (Figure 1A), a mutant virus that expressed a PTP that was nearly deficient in phosphatase activity (Supplementary Figure S1A). This mutagenesis was based on previous studies showing that mutation of cysteine 119 to serine (C119S) in the P-loop motif of the closely related PTP of *Autographa californica* NPV (AcMNPV) almost completely abolishes phosphatase activity [10], [12]. To our surprise BmPTP-C119S induced ELA in 5^th^ instar *B. mori* in a manner similar to that induced by wild-type BmNPV (Figure 1B). This indicated that the phosphatase activity of PTP is not required for the induction of ELA.

In order to determine whether the full-length PTP protein or RNAs transcribed from the *ptp* locus is required for the induction of ELA, we next generated BmPTP-Y9stop and BmPTP-E93stop (Figure 1A). These BmNPV mutants each carried a *ptp* gene with a point mutation in the coding region that generated a premature stop codon. These mutations likely had little effect on the structure of the expressed mRNAs, however, the expressed proteins were only 9 or 93 amino acid residues in length. In a manner similar to that observed with BmPTPD, BmPTP-Y9stop and BmPTP-E93stop were both unable to induce ELA in larval *B. mori* (Figure 1B). This indicated that the PTP protein itself is required for the induction of ELA but not mRNAs transcribed from the *ptp* gene.

### PTP binds ORF 1629, a WASP-like capsid protein

**Figure 2:**
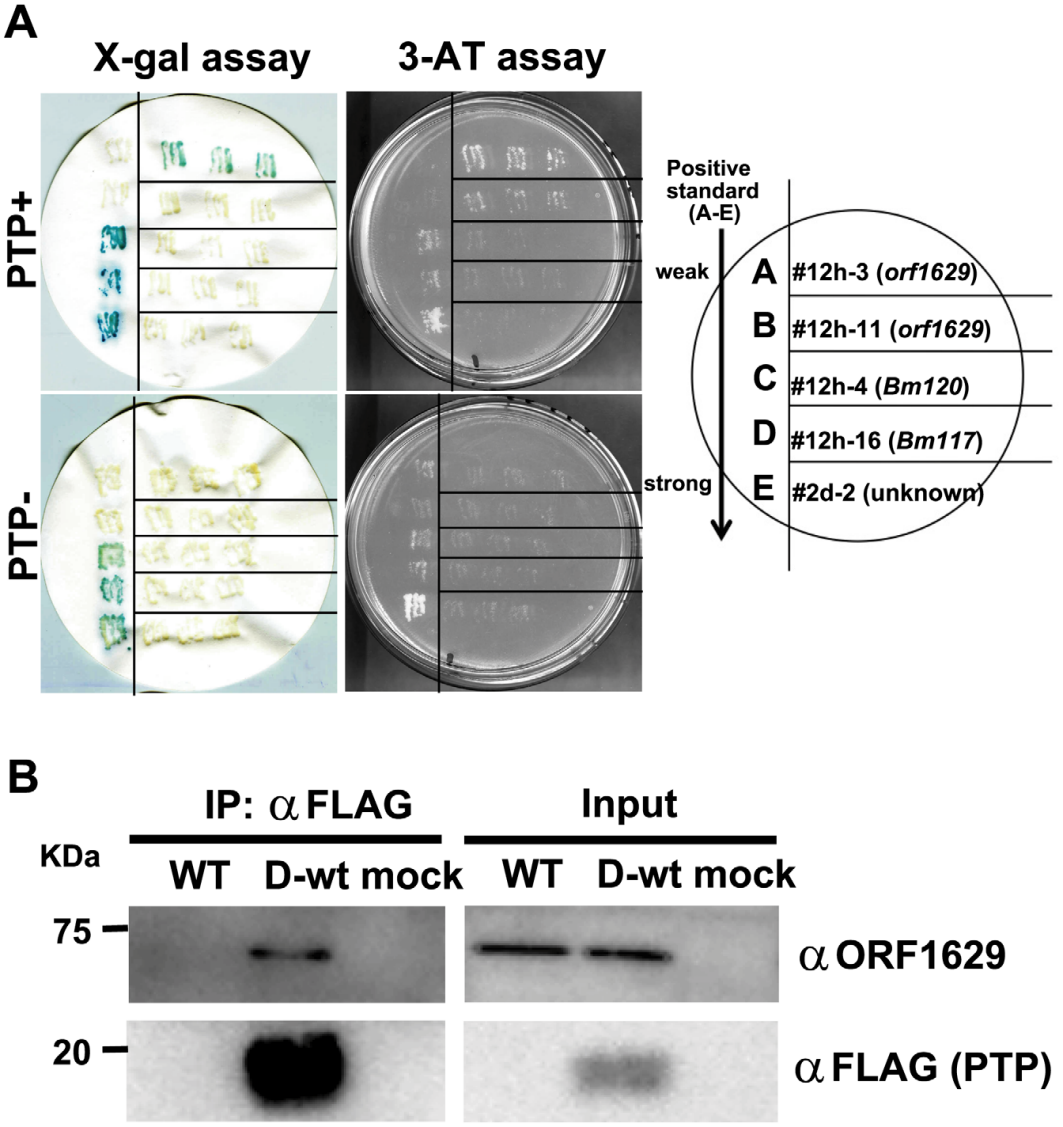
PTP interacts with ORF1629 in BmNPV-infected cells. (A) A yeast two-hybrid screen was performed to identify interactions between BmNPV PTP and proteins in BmNPV-infected (12 h p.i.) BmN cells or in epidermal tissues from BmNPV-infected (2 d p.i.) 5^th^ instar *B. mori*. This screening identified 5 PTP-interacting clones (12h-3, 12h-11, 12h-4, 12h-16, and 2d-2) by X-gal and 3-AT assays. Clones 12h-3, 12h-11, 12h-4, and 12h-16 were derived from BmNPV-infected BmN cells whereas clone 2d-2 was derived from BmNPV-infected larval *B. mori*. A legend showing the location of positive standards and PTP-interacting clones (streaked in triplicate) is shown to the right. (B) Interaction of PTP and ORF1629 in BmNPV-infected BmN cells. BmN cells were inoculated with BmNPV (WT) or BmPTPD-wt (D-wt) at an MOI of 5 or mock-infected (mock). BmPTPD-wt expresses FLAG-tagged PTP under an authentic *ptp* promoter. At 72 h p.i., the cells were harvested and immunoprecipitated with anti-FLAG antibody, and then subjected to western blot analysis (left panels) with anti-ORF1629 antibody or anti-FLAG antibody. The right “Input” panels show western blot analysis using whole cell extracts.

Our mutagenesis experiments indicated that the phosphatase activity of PTP is not required for the induction of ELA, thus we next used a yeast two-hybrid (Y2H) screening system to identify proteins that likely interact with PTP. The Y2H screening identified 5 clones which potentially interact with PTP (Figure 2A). Four of the clones (#12h-3, -4, -11, and -16) contained BmNPV-derived sequences whereas one clone (#2d-2) contained a *B. mori*-derived sequence of unknown function. The protein encoded by clone #12h-3 showed the strongest interaction with PTP (Figure 2A). This clone contained a nearly full-length (1572 nts) open reading frame (ORF) corresponding to ORF1629 of BmNPV (Supplementary Figure S2A). ORF1629 encodes a WASP-like protein that localizes at one end of the nucleocapsid structure [14], [15]. The deduced protein encoded by the clone #12h-11 corresponded to the C-terminal 347 amino acid residues (64% of full-length) of ORF1629 and exhibited a moderately strong interaction with PTP (Supplementary Figure S2A). Further analysis in yeast revealed that (i) the C-terminal of ORF1629 is critical for interaction with PTP (Supplementary Figure S2A), and (ii) the N-terminal 90 amino acid residues of PTP do not interact with ORF1629. The inability of the N-terminal of PTP to interact with ORF1629 is consistent with our locomotion assay results showing that BmPTP-E93stop did not induce ELA (Figure 1B).

In order to examine whether PTP interacts with ORF1629 in BmNPV-infected BmN cells, we next generated BmPTPD-wt. BmPTPD-wt is a derivative of BmPTPD that expresses FLAG-tagged PTP under an authentic *ptp* promoter immediately upstream of the *polh* gene (Supplementary Figure S3B–C). The authentic promoter of *ptp* was identified by 5′-RACE analyses (Supplementary Figure S3A). Immunoprecipitation experiments with anti-FLAG antibody and cell extracts from cells infected with BmPTPD-wt clearly showed that PTP interacts strongly with ORF1629 (Figure 2B), confirming the results of the Y2H experiments.

### PTP is a BV structural component localized in the virion envelope

**Figure 3:**
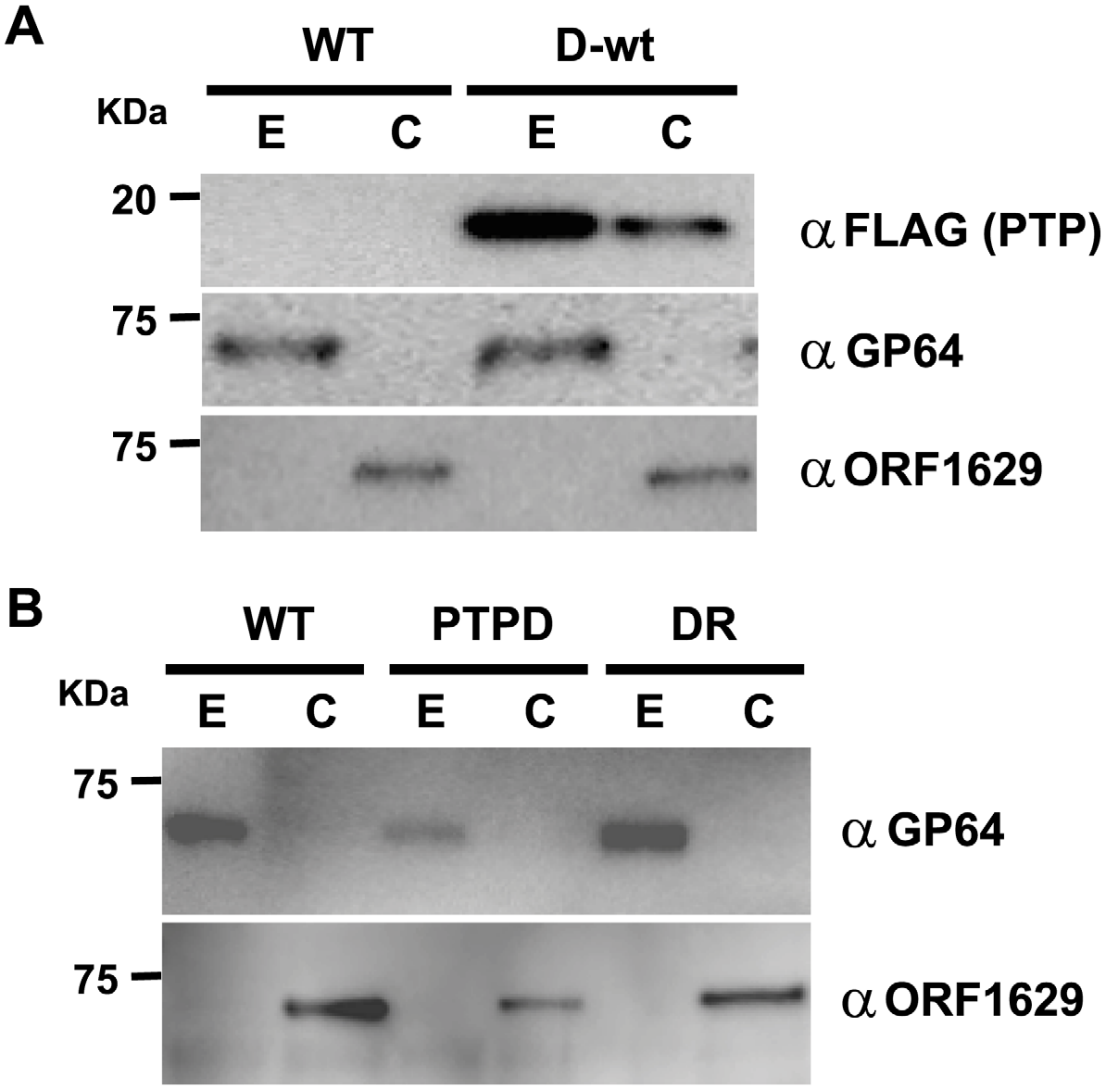
PTP is an envelope-associated protein required for the production of normal virions. (A) Localization of PTP in the envelope and capsid fractions of budded virus. Western blot analysis of envelope (E) and capsid (C) fractions of budded virus (BV) of BmNPV or BmPTPD-wt were performed with anti-FLAG, anti-GP64 or anti-ORF1629 antibodies. (B) Localization of GP64 and ORF1629 in PTP-deficient BV. Western blot analysis of envelope (E) and capsid (C) fractions of BV of BmNPV, BmPTPD, and BmPTPDR were performed with anti-GP64 or anti-ORF1629 antibody.

Because ORF1629 is a known structural protein, we speculated that PTP is also a structural protein that is associated with the BV envelope or capsid. Western blot analysis of BV-derived proteins that were fractionated into envelope and capsid components showed that PTP is primarily localized in the BV envelope (Figure 3A).

In order to examine whether the loss of PTP has any effects on the structural properties of the BV, we investigated the relative levels of ORF1629 and GP64 in BVs that were isolated from BmN cells infected with BmNPV, BmPTPD or BmPTPDR (a repair virus of BmPTPD) by western blot analysis. GP64 is a major envelope protein of BV that is essential for cell-to-cell infection [16]. The amounts of both GP64 and ORF1629 were reduced in the envelope and capsid, respectively, of BmPTPD BVs, in comparison to the corresponding BV fractions of BmNPV and BmPTPDR (Figure 3B). These findings indicated that disruption of *ptp* results in the formation of abnormal BVs with potentially reduced virus infectivity and/or replication.

### Loss of PTP leads to reduced progeny production in BmN cells and silkworm larvae

The role of PTP in the productive infection of BmNPV was investigated in BmN cells and silkworm larvae at 3 and 4 days postinfection (d p.i.), respectively. These time points were chosen because the production of BV and OB of wild-type BmNPV peak at these times. In BmN cells, BmPTPD produced about 50% fewer BVs and OBs in comparison to wild-type BmNPV at 3 d p.i. (Supplementary Figure S4). The reduction in BmPTPD OB production in BmN cells was consistent with that found in Sf9 cells infected with a *ptp*-deleted AcMNPV [17], [18]. Similar reductions in BV and OB production were also observed in BmN cells infected with BmPTP-Y9stop- and BmPTP-E93stop (Supplementary Figure S4). In contrast, BmPTP-C119S and BmPTPDR produced wild-type levels of BV and OB in BmN cells (Supplementary Figure S4). These results indicated that the expression of full-length PTP is required for the production of wild-type levels of BV and OB in BmN cells.

**Figure 4:**
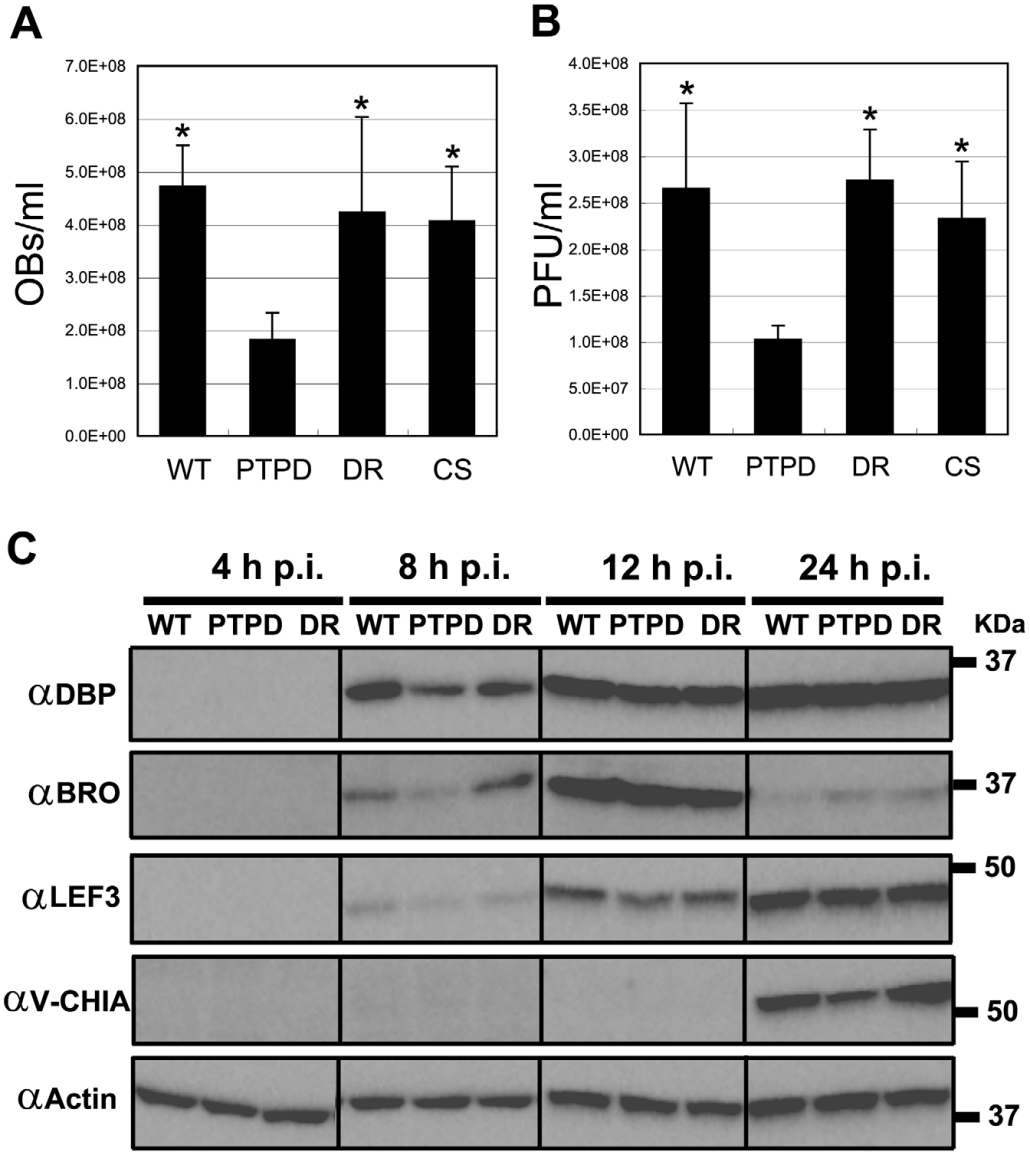
BmPTPD produces fewer progeny in 5^th^ instar *B. mori* and shows a delay in late gene expression in BmN cells. Production of OBs (A) and BVs (B) in the hemolymph of larvae infected with BmNPV, BmPTPD, BmPTPDR or BmPTP-C119S at 4 d p.i. Data shown are means ± standard deviation (SD) (N = 4). *p<0.05, one-way ANOVA with Tukey's post test in comparison to BmPTPD. (C) Western blot analysis of the expression of viral gene products in BmN cells infected with BmNPV, BmPTPD or BmPTPDR. The proteins were separated by SDS-PAGE, transferred to a nitrocellulose membrane, and immunoblotted with antibodies that recognize BmNPV early-expressed (DBP, BRO, and LEF3) or late-expressed (V-CHIA) proteins or actin. Similar results were obtained in two independent experiments. Abbreviations: WT, BmNPV; PTPD, BmPTPD; DR, BmPTPDR; and CS, BmPTPD-C119S.

We next examined if the dramatic drop in BV and OB production that was found in BmN cells also occurred in BmPTPD-infected silkworm larvae. The production of BV and OB in the hemolymph of BmPTPD-infected larvae was less than 50% of that found in wild-type BmNPV-infected larvae at 4 d p.i. (Figure 4A–B). In contrast, the production of BV and OB in larvae infected with BmPTP-C119S or BmPTPDR was similar to that found in BmNPV-infected larvae (Figure 4A–B). These *in vivo* findings were essentially identical to those found *in vitro* and suggested again that the PTP protein, but not its enzymatic activity, is essential for normal BV and ODV production. In addition, we did not observe significant differences in the median lethal dose (LD_50_) of BmNPV, BmPTPD, BmPTPDR, and BmPTP-C119S in 5^th^ instar *B. mori* (Supplementary Table S1), suggesting that the absence of PTP does not alter the virulence of BmNPV.

In order to investigate why BmPTPD produced fewer progeny, the expression profiles of a series of known baculovirus early and late gene products were examined by western blot analysis. Western blot analysis clearly showed that the expression of both early (DBP, BRO, and LEF3) and late (V-CHIA) proteins was delayed in BmPTPD-infected BmN cells (Figure 4C). These results indicated that loss of PTP caused a delay in the infection cycle, a delay that presumably led to the production of fewer BVs and OBs.

**Figure 5:**
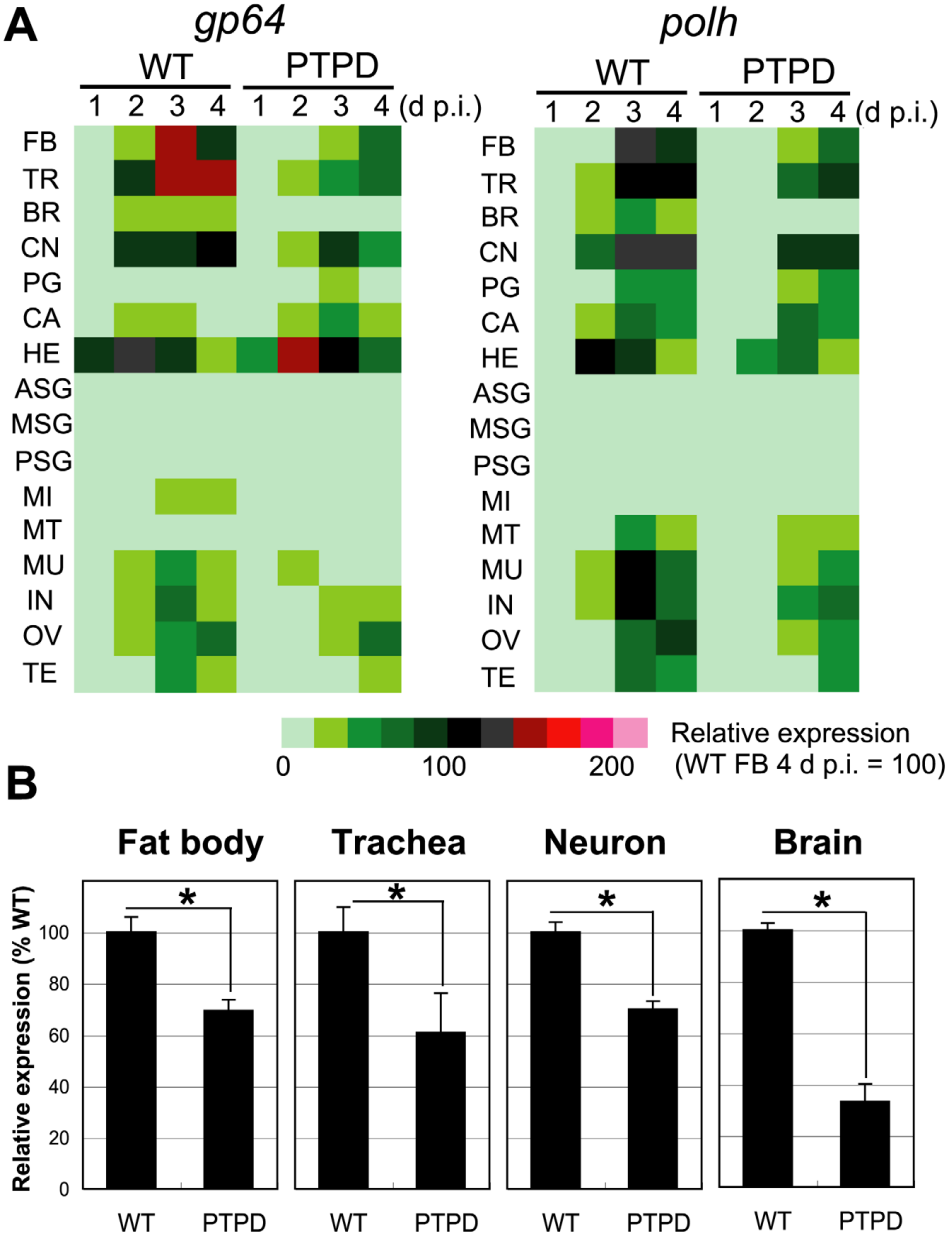
Reduced expression of viral genes in tissues of larvae infected with a *ptp*-disrupted virus. (A) Heatmaps of viral gene expression in 16 tissues of 5^th^ instar *B. mori* infected with BmNPV (WT) or BmPTPD (PTPD). The tissues were dissected from virus-infected larvae at 1, 2, 3, and 4 d p.i., and the expression of the early/late and very late genes *gp64* and *polh*, respectively, were quantified by qRT-PCR. Tissues from 5 to 30 larvae were mixed and used for the preparation of cDNAs. Abbreviations: FB, fat body; TR, trachea; BR, brain; CN, central nerve; PG, prothoracic gland; CA, corpora allata; HE, hemocyte; ASG, anterior silk gland; MSG, middle silk gland; PSG, posterior silk gland; MI, midgut; MT, Malpighian tubule; MU, muscle; IN, integument; OV, ovary; and TE, testis. (B) Expression of *polh* in fat body, trachea, central nerve, and brain. Tissues were dissected from four individual larvae at 4 d p.i. First strand cDNAs were generated from individual larvae and qRT-PCR was performed using primers that targeted the *polh* gene. Data shown are means ± SD (N = 4). *p<0.05, Student's t-test.

In order to examine the effects of *ptp* deletion in larval *B. mori* in greater detail, we measured the expression of viral early/late (*gp64*) and very late (*polh*) class genes in 16 tissues that were isolated from BmNPV- or BmPTPD-infected larvae. qRT-PCR analyses showed that, with the exception of a few tissues (i.e., corpora allata, prothoracic glands, and hemocytes), the relative expression levels of *gp64* and *polh* were much lower in BmPTPD-infected larvae than in BmNPV-infected larvae (Figure 5A). The expression of *polh* in the brain of BmPTPD-infected larvae at 4 d p.i. showed the most dramatic (67%) reduction (Figure 5B). These findings were consistent with the western blot analyses indicating that the replication cycle of BmPTPD was generally delayed but went further to show that the reduction in virus replication was most pronounced in the larval brain.

## Discussion

The manipulation of the behavior of caterpillars by baculoviruses has been known for over 100 years as *Wipfelkrankheit*. Recent developments in molecular biological and genomic tools have led to the identification of two baculovirus genes, *ptp* and *egt*, that are involved in altering host behavior [8], [9]. Interestingly, the baculovirus appears to have obtained both *ptp* and *egt* from an ancestral host. Hoover et al. [9] hypothesize that the *egt* gene product (EGT), a protein that is known to inactivate 20-hydroxyecdysone, controls the climbing behavior of NPV-infected gypsy moth larvae by hormonal regulation. On the other hand, the mechanistic action of how the *ptp* gene establishes behavioral control of host larvae remains elusive. In this study, we attempted to unravel this intriguing mystery by dissecting the functions of *ptp*/PTP in BmNPV-infected silkworms. We surprisingly found that the phosphatase activity of PTP appears not to be required for the behavioral control. In addition, we found that BmNPV induces ELA only when *ptp* mRNA is translated as a full-length protein, suggesting a non-enzymatic role for PTP. Our analyses revealed that PTP is a structural component of BV that is required for the production of mature BVs with full infectivity. We also found that loss of PTP dramatically reduces virus gene expression in several host tissues, especially in the brain.

Previous biochemical studies show that baculovirus PTP has the ability to remove phosphate groups from protein and RNA substrates [10]–[13]. In this study we confirmed that BmNPV PTP is also a functional phosphatase (Supplementary Figure S1A). Biologically, PTP and baculovirus LEF-4 have been predicted to play coordinated roles in 5′ cap formation of baculovirus late mRNAs [18]. A double-knockout mutant of *ptp* and *lef-4* of AcMNPV, however, does not show defects in mRNA cap formation and replicates normally in cultured cells [18]. Thus, the overall biological significance of the phosphatase activity of baculovirus PTP is still unknown. Interestingly, the *ptp* gene is conserved in Group I NPVs (e.g., BmNPV) but not in Group II NPVs (e.g., LdMNPV). Group II NPVs, however, are also able to induce ELA even though they lack the ability to produce PTP. This conundrum can be explained by the presence of the *egt* gene, a gene that is found in both Group I and II NPVs [4]. PTP and EGT appear to induce different types of ELA. PTP is involved in wandering-like ELA that is dramatically enhanced by light and shows positive phototropism [8], whereas EGT is involved in the induction of vertical climbing behavior [9]. Thus, the baculovirus-induced “wandering” and “climbing” behaviors appear to be regulated by different viral genes but appear to work in concert in Group I NPVs to improve transmission of the virus. In addition, Group I and Group II NPVs have unique BV envelope structures (e.g., Group I NPVs use GP64 as an envelope fusion protein for host cell attachment whereas in Group II NPVs use the F protein [19]). These structural differences may lead to unique tissue tropism and modes of ELA induction by Group I and Group II NPVs. Additionally, there may be other baculovirus genes that are involved in induction of ELA but their roles in ELA may be difficult to identify if they are essential for other viral functions or if host-derived genes can partially substitute for their functions.

Modern baculoviruses have apparently captured a number of essential and non-essential ‘auxiliary’ genes from ancestral host insects by horizontal gene transfer [5]. The authentic biological function of these captured genes or their products may be maintained, modified or lost in modern baculoviruses so that they confer selective advantages. The viral fibroblast growth factor (*vfgf*) gene is a clear example of a captured ancestral host gene whose authentic function has been maintained during evolution [20], [21]. The protein encoded by *vfgf*, vFGF, transmits its signaling via a host FGF receptor that, when activated, causes the migration of hemocytes to virus-infected tissues. vFGF is thus able to usurp the host's signaling pathway in order to recruit hemocytes which, following infection, can disseminate the virus to other tissues and increase systemic infection. BmNPV *ptp* is another example of a captured ancestral host gene [8]. In the case of BmNPV *ptp*, however, the biological importance of the PTP protein appears to have changed over time from a protein with enzymatic significance to one that has structural significance for establishing infection in larval tissues that are critical for the induction of ELA. In this study, we show that PTP binds strongly to ORF1629, a baculovirus structural protein that is phosphorylated during the infection cycle [22]. We hypothesize that in modern baculoviruses, the ability of PTP to bind ORF1629 or some other target became more important because of the role it plays in increasing virus transmission. In contrast, the ability of PTP to dephosphorylate a potential protein or RNA substrate appears not to be as important or perhaps taken over by a host phosphatase or only required when the virus has to replicate under unusual conditions. Alternatively, the PTP protein may have a dual function as a structural protein in the induction of ELA and as a phosphatase enzyme perhaps during earlier stages of infection, in specific tissues, or different host developmental stages. The *ptp* gene is thus the first example of a host-derived gene whose product is utilized by the modern baculovirus in a completely different manner from how it was likely utilized in the ancestral host.

Wandering is a normal ELA behavior that occurs towards the end of the last larval instar that causes caterpillars to search for an appropriate location to undergo metamorphosis [23]. Wandering behavior is regulated by a combination of internal (e.g., larval size, hormones) and external (e.g., photoperiod) processes. In larval *Manduca sexta* the brain exerts a net inhibitory influence that prevents wandering behavior during the caterpillar feeding stage [24], [25]. At the hormonal level, wandering is induced by exposure to the 20-hydroxyecdysone which causes the brain to become excitatory during the wandering stage. We hypothesize that baculovirus infection of caterpillar brain also leads to an excitatory state leading to the induction of the wandering-like ELA that we observe in BmNPV-infected silkworms. Electrophysiological studies of the locomotory patterns in the brain and subesophageal ganglion from baculovirus-infected larvae will allow us to understand what occurs in the central nervous system during virus-induced ELA. Our current hypothesis suggests that the baculovirus plays a direct role in the induction of ELA by infecting the brain. However, other more subtle factors such as baculovirus-induced changes in host energy metabolism, signal transduction, sensitivity to light or gravity, etc. may also play roles in the induction of the various types of ELA.

In conclusion, we show here that PTP functions to induce wandering-like ELA in baculovirus-infected caterpillars as a structural protein and likely not as an enzyme. Notably, we found that virus propagation was markedly reduced in brain tissues when *ptp* was deleted from the BmNPV genome. These results tell an amazing story of how the modern baculovirus has evolved to use a captured host gene in a different way from how it was likely used by the ancestral host. Collectively, we conclude that PTP augments baculovirus infection of the brain and possibly other tissues that play critical roles in the induction of ELA.

## Materials and Methods

### Insects, cell lines, and viruses

Larval *B. mori* were reared as described previously [26]. BmN (BmN-4) cells were cultured at 27°C in TC-100 medium supplemented with 10% fetal bovine serum [26]. The T3 strain of BmNPV was used as the wild-type virus. The construction of BmPTPD (a *ptp* deletion mutant) and BmPTPDR (a repair virus of BmPTPD), have been reported previously [8] (see Figure 1A). The titers of BmNPV and mutant BmNPVs were determined by plaque assay on BmN cells [26].

### Generation of BmPTP-C119S, BmPTP-Y9stop, BmPTP-E93stop, and BmPTPD-wt

BmNPV genomic DNA containing *ptp* and its flanking regions were cloned into pcDNA3.1(-) and used as a template to generate mutations in the *ptp* gene. Mutagenesis was performed by overlapping PCR [26] and confirmed by DNA sequencing. The resultant plasmids were transfected with *Bsu*36I-digested BmPTPD DNA (a *Bsu*36I restriction endonuclease site is uniquely found within the *lac*Z gene cassette of BmPTPD) into BmN cells using Lipofectin reagent (Invitrogen). Five days after transfection, the medium was collected and stored at 4°C until use. Three recombinant BmNPVs expressing PTP-C119S (BmPTP-C119S), PTP-Y9stop (BmPTP-Y9stop), and PTP-E93stop (BmPTP-E93stop) (Figure 1A) were isolated by the identification of plaques that did not express β-galactosidase [26]. The presence of the mutated *ptp* genes in these constructs was confirmed by polymerase chain reaction (PCR) using primers ptpF1 and ptp_B (Supplementary Table S2).

BmPTPD-wt, a repair mutant of BmPTPD that expresses a FLAG-tagged PTP under an authentic *ptp* gene promoter (inserted immediately upstream of the *polh* gene) was generated by a two step process. Firstly, the FLAG-tagged *ptp* gene driven by the authentic *ptp* gene promoter (identified by 5′-RACE) was amplified by PCR using BmNPV DNAs and primers ptpEPS1 and ptpEPS3 (Supplementary Table S2). The amplicon was inserted into the transfer vector pBmEPS1 [27], and the recombinant transfer plasmid was transfected with *Bsu*36I-digested BmNPV-abb [27] genomic DNA into BmN cells using Cellfectin reagent (Invitrogen) [28]. A recombinant BmNPV (T3-wt) expressing the FLAG-tagged PTP was plaque-purified by the identification of plaques that were OB-positive. In the second step, the authentic *ptp* gene of T3-wt was disrupted by transfection of T3-wt genomic DNA with a plasmid carrying a *lacZ* gene cassette flanked by *ptp* gene sequences [8] into BmN cells using Cellfectin reagent (Invitrogen). BmPTPD-wt (Supplementary Figure S3), a recombinant BmNPV expressing FLAG-tagged PTP under the authentic *ptp* gene promoter (but not expressing authentic PTP) was identified by the formation of plaques expressing β-galactosidase [29] and by PCR using the primer sets BmEPS_F1/BmEPS_R1 and ptpF2/ptpG2 (Supplementary Table S2). Expression of FLAG-tagged PTP by BmPTPD-wt was confirmed by western blot analysis with anti-FLAG antibody (Sigma).

### Locomotion assay

Locomotion assays were performed as reported previously with minor modifications [30]. Briefly, 5^th^ instar *B. mori* (24 larvae per treatment) were starved for several hours, injected with 50 µl of a viral suspension containing 1×10^5^ PFU, and returned to the artificial diet at 27°C. Infected larvae were photographed at 3 h intervals from 84 to 132 h postinfection (h p.i.). At each 3 h interval, the 24 infected larvae (separated into 4 groups of 6 larvae) were placed in the center of a piece of paper marked with concentric circles (the radius of each circle was 5 mm greater than the previous circle, with a maximum radius of 100 mm). Photographs were taken with a digital camera at 1 min intervals until 5 min after release. The coordinates of each larva, at the midpoint of the third and fourth abdominal segments, was determined at each time point after release using ImageJ software (Rasband WS (2006) ImageJ. Bethesda, Maryland: U. S. National Institutes of Health, rsb.info.nih.gov/ij/). The distance moved during each 1 min-long interval was determined and summed up to derive total locomotory distance in 5 min. The locomotory distance of dead larvae was designated as zero.

### Yeast two-hybrid screening

Yeast two-hybrid (Y2H) screening was performed using the PROQUEST two-hybrid system (Gibco BRL) as described previously [31]. The Y2H screening used a cDNA library that was generated from BmNPV-infected BmN cells as described previously [31], as well as a cDNA library that was constructed using mRNAs purified from epidermal tissues from BmNPV-infected larvae (2 d p.i.).

### Western blotting and immunoprecipitation

BmN cells were infected with BmNPV, BmPTPD, or BmPTPDR at an MOI of 5 and harvested at 48 h p.i. Biochemical fractionation of the BmN cells was performed as described previously [32]. Procedures for the isolation of BVs and fractionation of BV components were reported previously [33]. SDS-PAGE and western blotting were performed using anti-FLAG antibody, anti-GP64 antibody (Santa Cruz Biotechnology), anti-ORF1629 antibody [14] (a gift from George F. Rohrmann), anti-LEF3 antibody [34], [35] (a gift from Eric B. Carstens), anti-BRO antibody [36], anti-DBP antibody [37], and anti-actin antibody (Santa Cruz Biotechnology) as described previously [20]. Immunoprecipitation experiments were performed as described previously [20].

### OB and BV production in larval *B. mori*

Fifth instar *B. mori* (4 larvae per virus) were inoculated with virus and reared as described above. OBs that were released into the hemolymph, at 96 h p.i., were quantified from individual larva using a hemocytometer as described previously [26]. Hemolymph BV titer was determined by plaque assay on BmN cells [26].

### Quantitative reverse transcription-PCR (qRT-PCR)

Fifth instar *B. mori* were inoculated and reared as described above. Total RNA was prepared using Trizol reagent (Invitrogen) from 16 tissues (brain, corpora allata, central nerve, prothoracic gland, fat body, trachea, hemocyte, testis, ovary, anterior silk gland, middle silk gland, posterior silk gland, midgut, Malpighian tubule, integument, and muscle) that were dissected from BmNPV- or BmPTPD-infected, 5^th^ instar *B. mori* (5 to 30 larvae/tissue) at 1, 2, 3, and 4 d p.i. For the experiments shown in Figure 5B, total RNA was prepared using Trizol reagent from four tissues (brain, central nerve, fat body, and trachea) that were dissected from four individual 5^th^ instar larvae. First-strand cDNAs were synthesized from 0.2 µg of total RNA, and qRT-PCR was performed using Power SYBR Green PCR master mix (Applied Biosystems) using primers that were previously described [29]. PCR was performed using the StepOne real-time PCR system (Applied Biosystems) [29].

### Statistical analysis

Statistical analysis was performed using Prism 5 software (Graphpad). One-way analyses of variance (ANOVA) was performed with post hoc Tukey's test comparing each of the treatment group means with the mean of the control group. Locomotion assay data were subjected to Kruskal-Wallis analysis with post hoc Dunn's test. Student's t-test was used to compare values obtained in the qRT-PCR experiments (Figure 5B).

## Supporting Information

Figure S1 **PTP activity and confirmation of the genotype of the** ***ptp*** **gene mutants of BmNPV.** (A) Phosphatase activity of wild-type PTP (PTPwt) and C119S mutant PTP (PTPcs). PTPwt and PTPcs were expressed in *Escherichia coli*, and phosphatase activity was assessed using poly(Glu-Tyr) as the substrate. *p<0.05, Student's t-test. (B) Confirmation of the genotype of wild-type and mutant BmNPVs using primers ptpF1 and ptp_B (primer sequences are shown in **Supplementary** Table S2).
(PDF)

Figure S2 **Analysis of the interaction between PTP and ORF1629 using the yeast two-hybrid system.** (A) Identification of PTP-interacting domains of ORF1629. A schematic representation of authentic ORF1629 is shown by the top bar. The amino acid locations of the proline-rich, WASP-homology2, and connector/acid domains of ORF1629 are shown above the schematic representation. The results of the yeast two-hybrid X-gal screening assay are shown to the right. The + or − indicates a positive or negative interaction, respectively, between PTP and the indicated region of ORF1629. (B) Identification of ORF1629-interacting domains of PTP. A schematic representation of PTP is shown by the top bar. The results of the X-gal screening assay are shown to the right. The + or − indicates a positive or negative interaction, respectively, between ORF1629 and the indicated region of PTP.
(PDF)

Figure S3 **Construction of BmPTPD-wt.** (A) Determination of the transcriptional start sites of *ptp*. 5′-RACE analysis was performed using cDNAs prepared from BmNPV-infected BmN cells at 4 or 12 h p.i. The time post infection and number of independent clones that were obtained is shown in parentheses. (B) Schematic representation of BmPTPD-wt. The approximate locations of two pairs of PCR primers (BmEPS_F/R and ptpF2/G2) that were used in the genotyping experiments are shown. (C) Confirmation of the genotype of BmNPV (WT), BmPTPD (PTPD), and BmPTPD-wt (D-wt) by PCR with primer pairs BmEPS_F/BmEPS_R and ptpF2/ptpG2.
(PDF)

Figure S4 **BmN cells infected with** ***ptp*** **gene disrupted BmNPV mutants show reduced OB and BV production.** (A) Light microscopic observations of representative virus-infected BmN cells at 3 d p.i. (B) OB production in virus-infected BmN cells at 3 d p.i. (C) BV production in virus-infected BmN cells at 1, 2, and 3 d p.i. as determined by plaque assay on BmN cells In A, B, and C, the BmN cells were infected with virus at an MOI of 5. In B and C, the data shown are mean ± SD (N = 3). *p<0.05, one-way ANOVA, Tukey's post tests in comparison to BmPTPD. Abbreviations: WT, BmNPV; PTPD, BmPTPD; DR, BmPTPDR; CS, BmPTP-C119S; Y9, BmPTP-Y9stop; and E93, BmPTP-E93stop.
(PDF)

Table S1 **Dose-mortality of BmNPV, BmPTPD, BmPTPDR and BmPTP-C119S in 5^th^ instar** ***B. mori*** **.**
(PDF)

Table S2 **Primers used in this study.**
(PDF)

Text S1 Materials and Methods for Supplementary Information (PDF)
