# Supplementary figures and images for: The Baculovirus Uses a Captured Host Phosphatase to Induce Enhanced Locomotory Activity in Host Caterpillars
Source: PLoS Pathog. 2012 Apr 5;8(4):e1002644. doi: 10.1371/journal.ppat.1002644 (PMC3320614; doi:10.1371/journal.ppat.1002644)

**A**

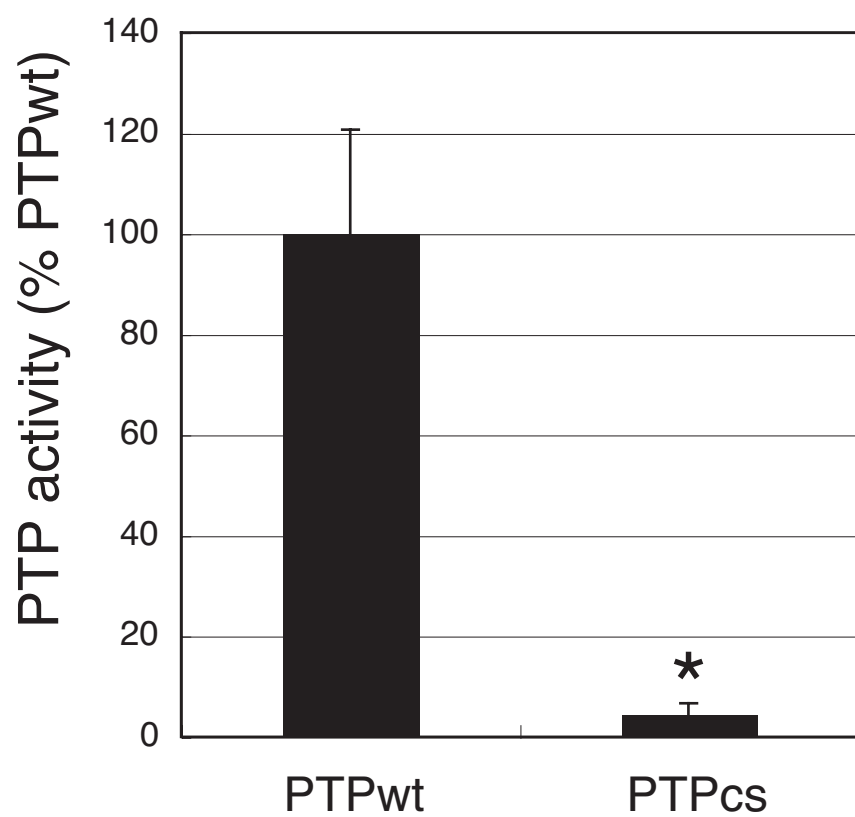

**B**

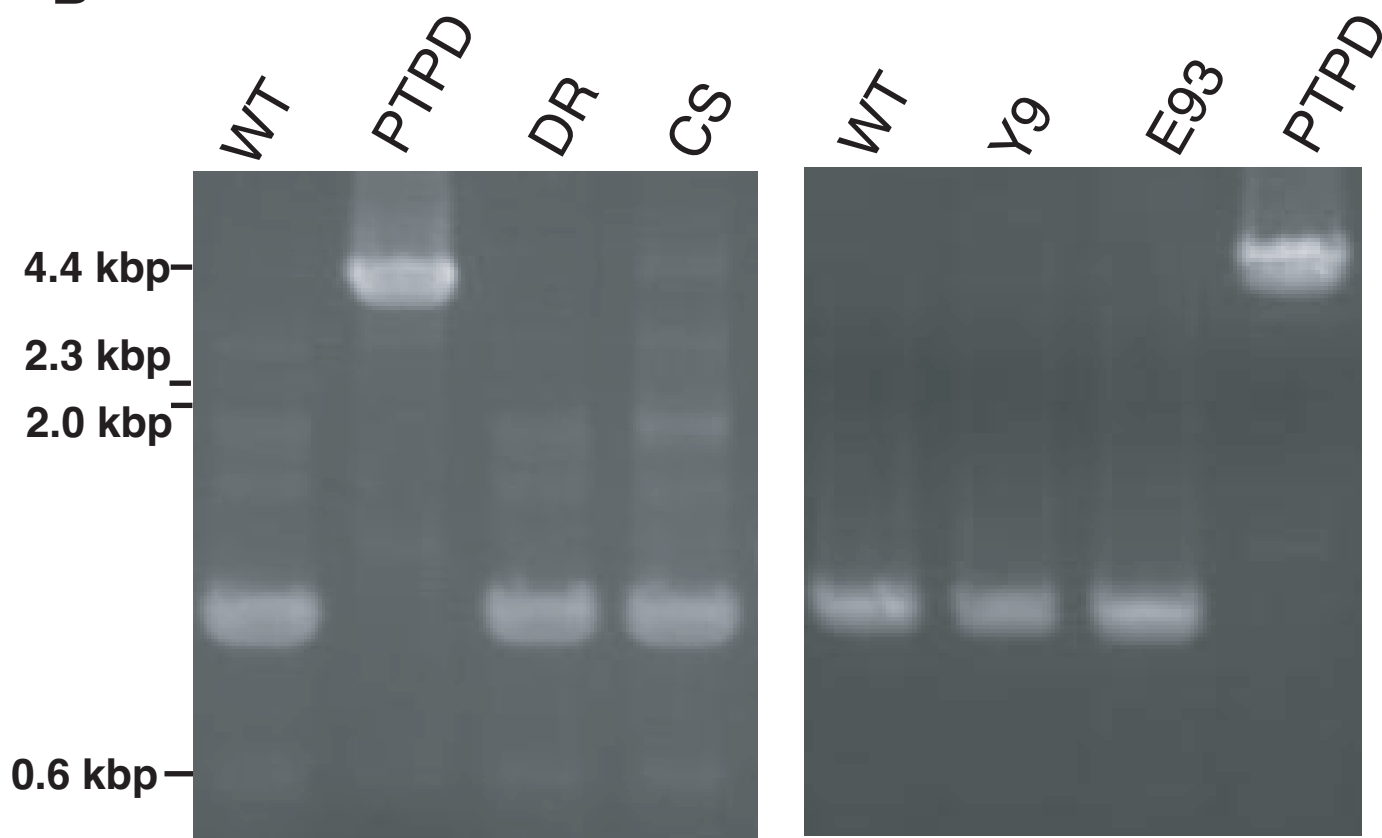

Supplement: Figure S1 — PTP activity and confirmation of the genotype of the ptp gene mutants of BmNPV. (A) Phosphatase activity of wild-type PTP (PTPwt) and C119S mutant PTP (PTPcs). PTPwt and PTPcs were expressed in Escherichia coli, and phosphatase activity was assessed using poly(Glu-Tyr) as the substrate. *p<0.05, Student's t-test. (B) Confirmation of the genotype of wild-type and mutant BmNPVs using primers ptpF1 and ptp_B (primer sequences are shown in Supplementary Table S2). (PDF) [file ppat.1002644.s001.pdf]

A

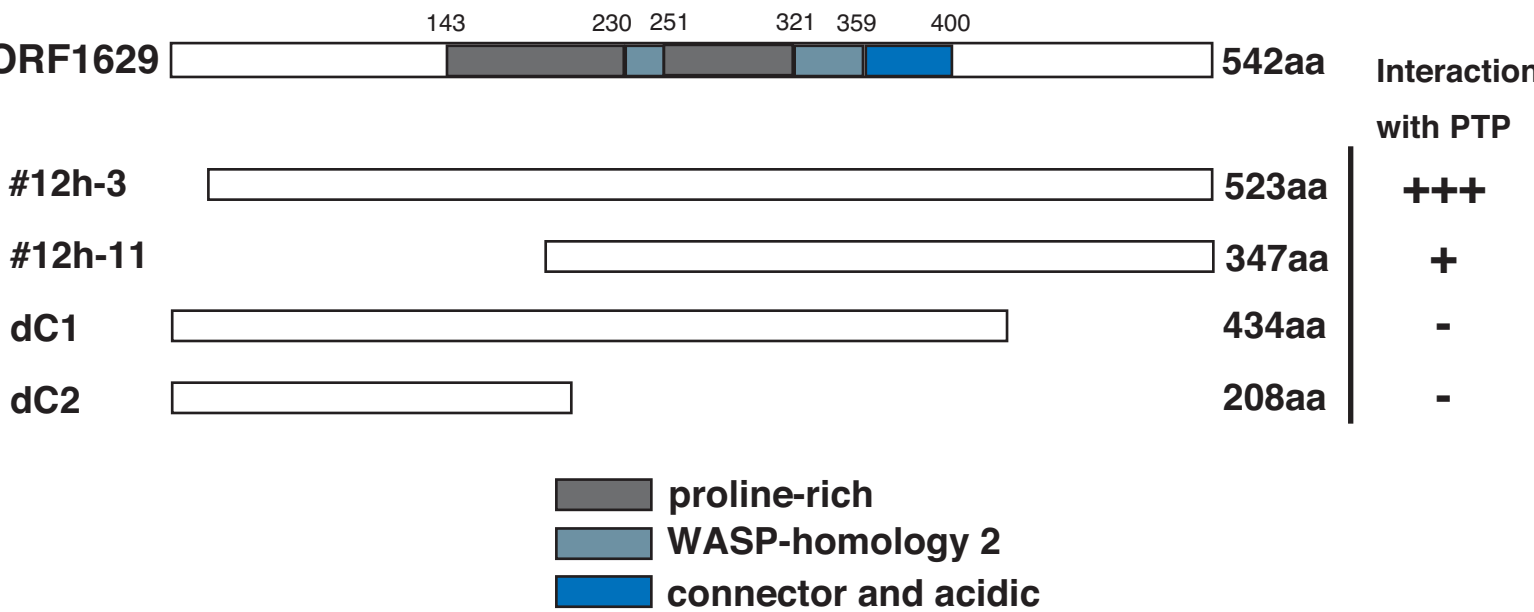

B

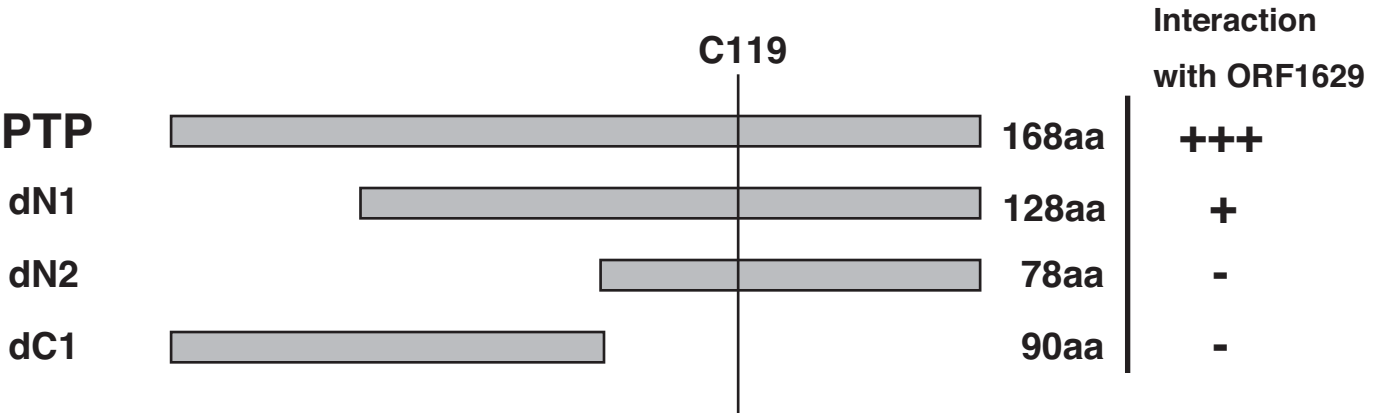

Supplement: Figure S2 — Analysis of the interaction between PTP and ORF1629 using the yeast two-hybrid system. (A) Identification of PTP-interacting domains of ORF1629. A schematic representation of authentic ORF1629 is shown by the top bar. The amino acid locations of the proline-rich, WASP-homology2, and connector/acid domains of ORF1629 are shown above the schematic representation. The results of the yeast two-hybrid X-gal screening assay are shown to the right. The + or − indicates a positive or negative interaction, respectively, between PTP and the indicated region of ORF1629. (B) Identification of ORF1629-interacting domains of PTP. A schematic representation of PTP is shown by the top bar. The results of the X-gal screening assay are shown to the right. The + or − indicates a positive or negative interaction, respectively, between ORF1629 and the indicated region of PTP. (PDF) [file ppat.1002644.s002.pdf]

**A**

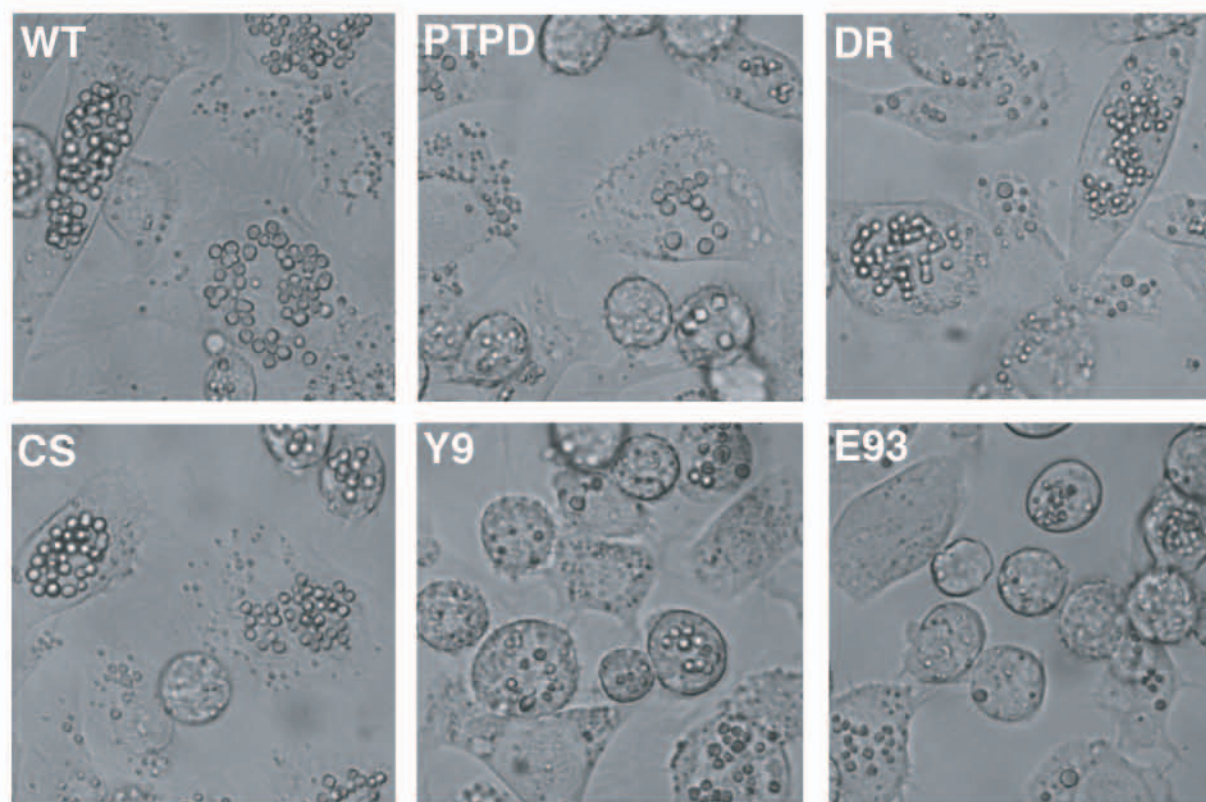

**B**

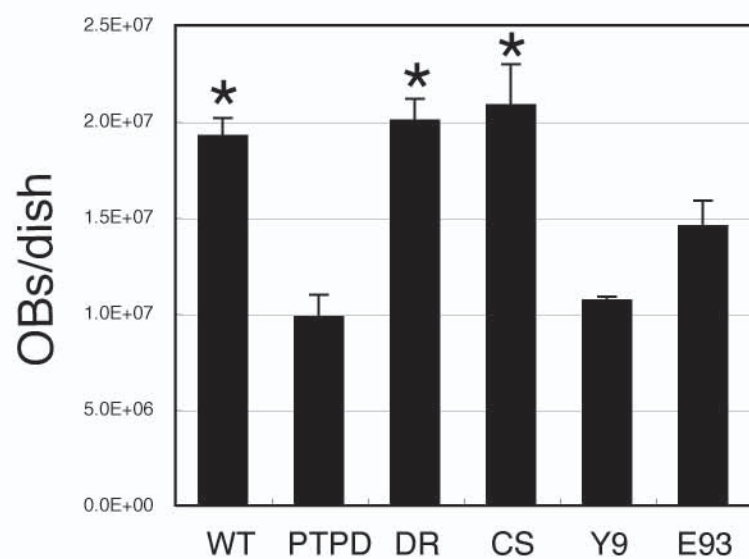

**C**

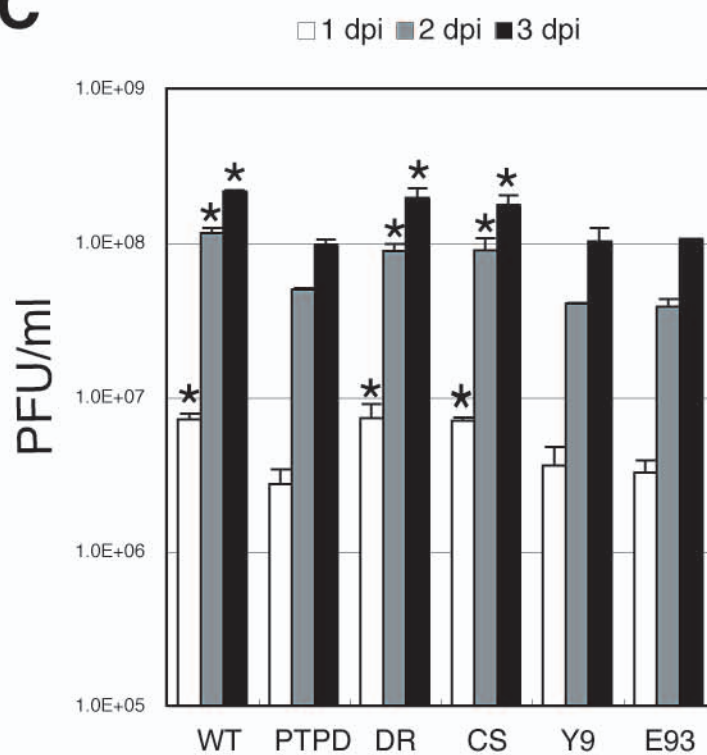

Supplement: Figure S4 — BmN cells infected with ptp gene disrupted BmNPV mutants show reduced OB and BV production. (A) Light microscopic observations of representative virus-infected BmN cells at 3 d p.i. (B) OB production in virus-infected BmN cells at 3 d p.i. (C) BV production in virus-infected BmN cells at 1, 2, and 3 d p.i. as determined by plaque assay on BmN cells In A, B, and C, the BmN cells were infected with virus at an MOI of 5. In B and C, the data shown are mean ± SD (N = 3). *p<0.05, one-way ANOVA, Tukey's post tests in comparison to BmPTPD. Abbreviations: WT, BmNPV; PTPD, BmPTPD; DR, BmPTPDR; CS, BmPTP-C119S; Y9, BmPTP-Y9stop; and E93, BmPTP-E93stop. (PDF) [file ppat.1002644.s004.pdf]
