# Supplementary material for: The Baculovirus Uses a Captured Host Phosphatase to Induce Enhanced Locomotory Activity in Host Caterpillars
Source: PLoS Pathog. 2012 Apr 5;8(4):e1002644. doi: 10.1371/journal.ppat.1002644 (PMC3320614; doi:10.1371/journal.ppat.1002644)

Katsuma et al., Fig. S3

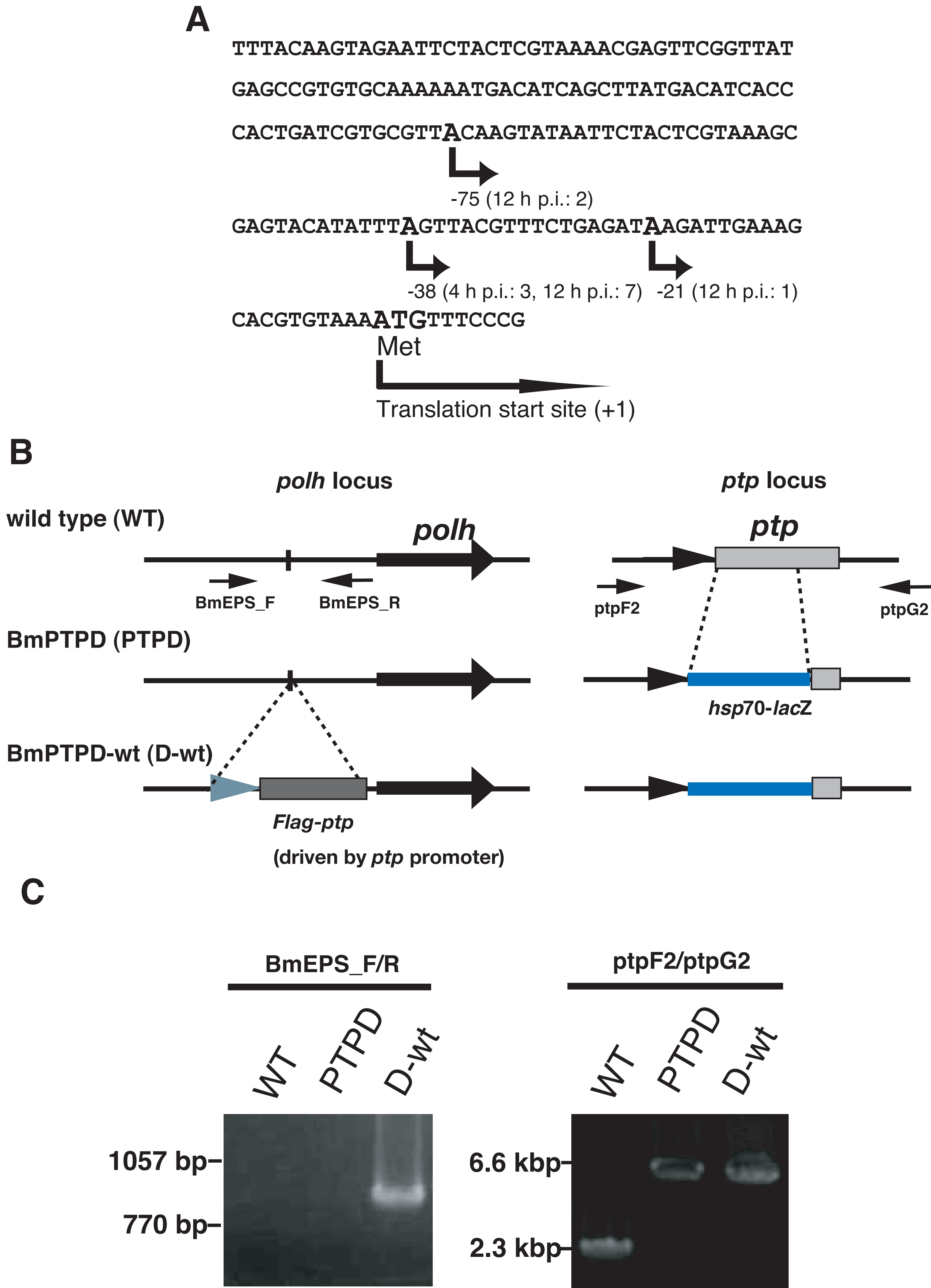

Supplement: Figure S3 — Construction of BmPTPD-wt. (A) Determination of the transcriptional start sites of ptp. 5′-RACE analysis was performed using cDNAs prepared from BmNPV-infected BmN cells at 4 or 12 h p.i. The time post infection and number of independent clones that were obtained is shown in parentheses. (B) Schematic representation of BmPTPD-wt. The approximate locations of two pairs of PCR primers (BmEPS_F/R and ptpF2/G2) that were used in the genotyping experiments are shown. (C) Confirmation of the genotype of BmNPV (WT), BmPTPD (PTPD), and BmPTPD-wt (D-wt) by PCR with primer pairs BmEPS_F/BmEPS_R and ptpF2/ptpG2. (PDF) [file ppat.1002644.s003.pdf]
