# Supplementary material for: The Baculovirus Uses a Captured Host Phosphatase to Induce Enhanced Locomotory Activity in Host Caterpillars
Source: PLoS Pathog. 2012 Apr 5;8(4):e1002644. doi: 10.1371/journal.ppat.1002644 (PMC3320614; doi:10.1371/journal.ppat.1002644)
Supplement: Table S1 — Dose-mortality of BmNPV, BmPTPD, BmPTPDR and BmPTP-C119S in 5th instar B. mori . (PDF) [file ppat.1002644.s005.pdf]

Table S1

Dose-mortality of BmNPV, BmPTPD, BmPTPDR and BmPTP-C119S in *B. mori* larvae

B. mori and viruses	LD_{50} (PFU)	95% Fiducial limit	
		Lower	Upper
WT	0.11	0.05	0.30
BmPTPD	0.09	0.05	0.13
BmPTPDR	0.15	0.08	0.17
BmPTP-C119S	0.07	0.02	0.14
