# Supplementary material for: The Baculovirus Uses a Captured Host Phosphatase to Induce Enhanced Locomotory Activity in Host Caterpillars
Source: PLoS Pathog. 2012 Apr 5;8(4):e1002644. doi: 10.1371/journal.ppat.1002644 (PMC3320614; doi:10.1371/journal.ppat.1002644)
Supplement: Table S2 — Primers used in this study. (PDF) [file ppat.1002644.s006.pdf]

Table S2. Primers used in this study

Primer	Sequence	Purpose
ptpF1	5'- ATATTTAGTTACGTTTCTGAG -3'	genotyping of recombinant viruses
ptp_B	5'- AAGGATCCCATCGCAATTAAGGACAAGC -3'	
BmEPS_F	5'- TGTCCGTTTGCTGGCAACTG -3'	
BmEPS_R	5'- TACTTATTTATTTGCGAGATGG -3'	
ptpF2	5'- TTCGCTCACTCGTCTATTCG -3'	
ptpG2	5'- AAGACGCTGTAAGCAGACAC -3'	
ptpEPS1	5'- AAGAATTCGTTCGGTTATGAGCCGTGTG -3'	Construction of BmPTPD-wt
ptpEPS3	5'- AACTGCAGATAGAATAAAGTATTTGTTAAAAAATGCAAATAATATTAATTACTTATCGTCGTCATCCTTGTAATCAATTAATAAATCTTGAACGT -3'	
ptpF1	5'- ATATTTAGTTACGTTTCTGAG -3'	qPCR for *ptp*
ptpR1	5'- TTAAATATCTGCACACCATG -3'	
