# Supplementary material for: The Baculovirus Uses a Captured Host Phosphatase to Induce Enhanced Locomotory Activity in Host Caterpillars
Source: PLoS Pathog. 2012 Apr 5;8(4):e1002644. doi: 10.1371/journal.ppat.1002644 (PMC3320614; doi:10.1371/journal.ppat.1002644)
Supplement: Text S1 Materials and Methods for Supplementary Information — (PDF) [file ppat.1002644.s007.pdf]

<u>**Supplementary Information**</u>

Materials and Methods

Phosphatase assay.

His-tagged wild-type PTP and a C119S mutant PTP were expressed in bacteria and purified as described previously [1]. Phosphatase activity was assessed using a Universal Tyrosine Phosphatase Assay kit (TAKARA BIO INC.) according to the supplier's protocol.

Larval bioassays.

The LD_{50} of BV was determined in 5th instar larvae by intrahemocoelic injection with various doses of BV diluted in TC-100 medium. Larvae were inoculated within 12 h after molting to the 5th instar. At least 20 larvae per dose were used in each of the experiments. Virus titers in hemolymph of infected larvae were determined by plaque assay on BmN cells.

5'-rapid amplification of cDNA ends (5'-RACE).

5'-RACE analysis to determine the transcriptional start sites of *ptp* was performed using a GeneRacer kit (Invitrogen) as described previously [2]. Total RNA was isolated with Trizol reagent from BmNPV-infected BmN cells at 4 or 12 h p.i. Temporal expression analysis using primers ptpF1 and ptpR1 (Table S2) first identified *ptp* mRNAs at 4 h p.i. First-strand cDNAs were synthesized from 5 μg of total RNA. Amplicons from the RACE reactions were cloned into pGEM-T Easy Vector (Promega), and DNA sequences were determined using an ABI Prism 3100 DNA sequencer (Applied Biosystems).

OB and BV production in BmN cells.

Viral replication in BmN cells was determined following the inoculation of BmN cells with virus at an MOI of 5. Following incubation for 1 h, the virus-containing culture medium was removed and fresh medium was added (0 h p.i.). A small amount of the culture medium was harvested at specific time points (1, 2, and 3 dpi) and viral titers were determined by plaque assay on BmN cells as described previously [3]. OBs were

isolated from BmN cells that were infected with BmNPV or mutant BmNPVs at 72 h p.i. and quantified as described previously [3].

References

1. Meng, Y., Katsuma, S., Daimon, T., Banno, Y., Uchino, K., Sezutsu, H., Tamura, T., Mita, K. & Shimada, T. The silkworm mutant *lemon* (*lemon lethal*) is a potential insect model for human sepiapterin reductase deficiency. *J. Biol. Chem.* **284**, 11698-11705 (2009).

2. Katsuma, S. & Shimada, T. *Bombyx mori* nucleopolyhedrovirus ORF34 is required for efficient transcription of late and very late genes. *Virology* 392, 230-237 (2009).

3. Nakanishi, T., Goto, C., Kobayashi, M., Kang, W. K., Suzuki, T., Dohmae, N., Matsumoto, S., Shimada, T. & Katsuma, S. Comparative studies of a lepidopteran baculovirus-specific protein FP25K: Development of a novel BmNPV-based vector with a modified *fp25K* gene. *J. Virol.* **84**, 5191-5200 (2010).
